# Characterisation of aptamer-anchored poly(EDMA-*co*-GMA) monolith for high throughput affinity binding

**DOI:** 10.1038/s41598-019-50862-1

**Published:** 2019-10-10

**Authors:** Caleb Acquah, Yi Wei Chan, Sharadwata Pan, Lau Sie Yon, Clarence M. Ongkudon, Haobo Guo, Michael K. Danquah

**Affiliations:** 1 Department of Chemical Engineering, Curtin University, Sarawak, 98009 Malaysia; 2 School of Nutrition Science, Faculty of Health Science, University of Ottawa, K1N 6N5 Ontario, Canada; 3 Biotechnology Research Institute, Universiti Malaysia Sabah, Kota Kinabalu, Sabah 88400 Malaysia; 4 School of Life Sciences Weihenstephan, Technical University of Munich, Freising, 85354 Germany; 5 Department of Computer Science and Engineering, University of Tennessee, Chattanooga, TN 37403 United States; 6 SimCenter, University of Tennessee, Chattanooga, TN 37403 United States; 7 Department of Chemical Engineering, University of Tennessee, Chattanooga, TN 37403 United States

**Keywords:** Biophysical chemistry, Bioanalytical chemistry

## Abstract

Immobilisation of aptameric ligands on solid stationary supports for effective binding of target molecules requires understanding of the relationship between aptamer-polymer interactions and the conditions governing the mass transfer of the binding process. Herein, key process parameters affecting the molecular anchoring of a thrombin-binding aptamer (TBA) onto polymethacrylate monolith pore surface, and the binding characteristics of the resulting macroporous aptasensor were investigated. Molecular dynamics (MD) simulations of the TBA-thrombin binding indicated enhanced Guanine 4 (G4) structural stability of TBA upon interaction with thrombin in an ionic environment. Fourier-transform infrared spectroscopy and thermogravimetric analyses were used to characterise the available functional groups and thermo-molecular stability of the immobilised polymer generated with Schiff-base activation and immobilisation scheme. The initial degradation temperature of the polymethacrylate stationary support increased with each step of the Schiff-base process: poly(Ethylene glycol Dimethacrylate-co-Glycidyl methacrylate) or poly(EDMA-co-GMA) [196.0 °C (±1.8)]; poly(EDMA-co-GMA)-Ethylenediamine [235.9 °C (±6.1)]; poly(EDMA-co-GMA)-Ethylenediamine-Glutaraldehyde [255.4 °C (±2.7)]; and aptamer-modified monolith [273.7 °C (±2.5)]. These initial temperature increments reflected in the associated endothermic energies were determined with differential scanning calorimetry. The aptameric ligand density obtained after immobilisation was 480 pmol/μL. Increase in pH and ionic concentration affected the surface charge distribution and the binding characteristics of the aptamer-modified disk-monoliths, resulting in the optimum binding pH and ionic concentration of 8.0 and 5 mM Mg^2+^, respectively. These results are critical in understanding and setting parametric constraints indispensable to develop and enhance the performance of aptasensors.

## Introduction

Biosensors utilise specific bioprobes to detect and analyse target molecules^1–3^. They have garnered considerable attention in research and applications for medical diagnosis and prognosis, as well as the detection of environmental contaminants such as pesticides and heavy metals^4,5^. Unlike conventional cellular and biochemical methods, bioaffinity sensing is largely devoid of long sample processing times, offer specific and selective target binding, and can be relatively cost-effective to conceive^6^. A range of options are available in the context of bioaffinity probes, including antibodies, enzymes, cells, aptamers, amongst others, for the development of biosensors. In general, antibodies are the most utilised bioaffinity probes in the design of biosensors, and are uniquely referred to as immunosensors. However, the development and application of antibodies as bioaffinity probes, are often challenged by ethical issues, short shelf-life and high production costs, along with a myriad of additional factors such as binding specificity, bioavailability, immunogenicity and thermal stability^7–9^.

On this front, aptamers have garnered widespread attention towards alleviating these challenges to a considerable extent. Aptamers are short, single stranded oligonucleotides that can be chemically synthesised to target a wide range of proteins, cells, lipids and ions. These cognate targets are popularly known as ‘apatopes’. Aptamer-target interactions are non-covalent and are based on any or a combination of the following: electrostatic, hydrogen bonding, aromatic stacking, hydrophobic, and Van der Waals interactions^7,10^. Aptamers are developed through a robust iterative process known as Systematic Evolution of Ligands by Exponential enrichment (SELEX)^11–13^, which inherently endow them with laudable attributes like better binding strength and specificity compared to antibodies^14^. They also possess other benefits concerning manufacturing, biophysical and biochemical attributes, including a large array of target space, low production costs, thermal and chemical stability, low to negligible ethical issues, prolonged shelf-life/reusability and uncomplicated pre-/post biomodification mechanisms^15–17^.

Molecular interactions between aptamers and their targets are affected by physicochemical conditions of the binding environment, including ionic concentration, pH, type and characteristics of the support matrix, aptamer modifications, and temperature^18,19^. The immobilisation of aptamers to form aptasensors enhances their recycling through successive regeneration for continuous flow applications^20,21^. In recent times, the immobilisation of aptamers on macroporous monolithic matrices, with convective flow characteristics for high throughput biosensing and bioseparation applications, have emerged as a major research endeavour^22–26^. Polymethacrylate monoliths, due to their tunable macroporous structure, biocompatibility and simple functionalisation chemistries, have gained interest to be envisaged as the synthetic polymer core, for developing the next generation of biosensors for high throughput applications^27,28^. A few instances of functionalisation chemistries, compatible with polymethacrylate monoliths, for molecular coupling of aptamers, include streptavidin-biotin, epoxy-based, glutaraldehyde, disuccinimidyl carbonate, azalactone, thiolene click, and carbonyldiimidazole^27^. Molecular coupling of aptamers on polymethacrylate monoliths, through Schiff-base chemistry, ensures covalently bonded interactions between the monolith and aptamer^22^, thereby reducing the likelihood of potential leaching of the aptameric ligand. Furthermore, attributing to the fact that it incoporates a spacer-arm through amine-aldehyde linking groups to prevent the occurrence of steric hindrance, Schiff-base chemistry was selected as the molecular coupling chemistry in this study.

Although the suitability of various coupling chemistries for the development of aptamer-modified monoliths has been inexhaustibly investigated by only a handful of past studies, none has focused on probing the impact of biophysical and biochemical parameters on the binding characteristics and performance. Specifically, a study investigating the convective mass transfer and flow hydrodynamic properties of disk polymethacrylate monoliths and the high specificity binding characteristcis of aptameric ligands, to develop smart biosensing formats, is lacking in the literature. While targeting this lacuna, the central objectives of the current investigation stands two-fold: to synthesise and evaluate the chromatographic binding performance of the aptamer-modified poly(EDMA-co-GMA) disk-monoliths under varying physicochemical conditions; and to study the impact of key physicochemical parameters on the surface charge distribution of the aptamer-modified monolith. The present work reports the molecular anchoring of a thrombin binding aptameric ligand on a disk polymethacrylate monolith, including molecular dynamic simulation and analysis of its physicochemical characterisations.

## Experimental Methodology

### Materials

15-mer thrombin binding DNA aptamers (TBA) modified with an amino moiety coupled with a C6 spacer arm (5AmMC6) was synthesised by Base pair biotechnologies (Malaysia). Primary sequence of synthesised aptamer is 5′-/5AmMC6/GGT TGG TGT GGT TGG-3′. The following chemicals were purchased from Sigma-Aldrich (USA): ethylene glycol dimethacrylate, EDMA, (MW 198.22, 98%); glycidyl methacrylate, GMA, (MW 142.15, 97%); methanol (HPLC grade, MW 32.04, 99.93%); azobisisobutyronitrile, AIBN, (MW 164.21 g/mol, 98%); cyclohexanol (MW 100.16, 99%); hydrochloric acid (HCl, MW 36.5, 37%); phosphate buffer solutions (PBS); Trizma HCl (MW 157.60, 99%); ethylenediaminetetraacetic acid (MW 292.24, 99%); sodium cyanoborohydride; ethylenediamine, (MW 62.84, 95%); EDA, (MW 60.10, 99%); glutaraldehyde, GA, (MW 100.12, 25%); and human alpha thrombin.

### Methods

#### Molecular dynamics simulation

Molecular dynamics (MD) simulation of the stability of 15-mer TBA with thrombin in the microenvironment was studied using Nanoscale Molecular Dynamics (NAMD)^29^ with the Chemistry at HARvard Molecular Mechanics (CHARMM) force field^30^. Transferable intermolecular potential with 3 points (TIP3P)^31^ model was used to describe the aqueous solution at an ionic strength of 100 mM NaCl. This system comprises 71,444 atoms. MD simulation was carried out in a 10.5×86.8×82.8 nm^3^ water box under the NPT (1 atm, 300 K) ensemble.

#### Synthesis of polymethacrylate monoliths

Disk polymethacrylate monoliths were synthesised *in situ* via free radical polymerisation, as reported previously by our group^26,32,33^. Briefly, 0.5 mL of monoliths were prepared using 60/40% v/v of monomer to porogen composition. The monomeric composition constituted 60% v/v GMA as the functional monomer and 40% v/v EDMA as the cross-linker. Cyclohexanol was used as the porogen, and the polymerisation mixture was sonicated for 10 mins. The mixture was then transferred to a 1.5 cm I.D BIORAD polypropylene column and sparged with nitrogen for about 10 mins. The column was sealed thereafter and polymerisation commenced isothermally at a set point temperature of 65 °C for 16 h. The fabricated disk polymethacrylate monoliths were washed with methanol followed by deionised water, using the NGC Discover chromatography (Next Generation Chromatography Discover 100 Chromatography system, BIORAD, Melbourne, Australia) system, until a constant baseline was obtained over an extended period of time. The washed disk-monoliths were stored under wet conditions at 4 °C, for activation and functionalisation.

#### Aptamer immobilisation

Prior to activation and functionalisation, the disk-monoliths were incubated at 60 °C to remove bubbles trapped within the pores of the adsorbent. Thrombin binding aptamer stock solutions of 100 µM were prepared with phosphate buffer A (10 mM phosphate buffer + 20 mM potassium chloride + 137 mM sodium chloride + 5 mM MgCl_2_ at pH 7.4) and stored at −20 °C. Aptamer immobilisation was performed by recirculation of aptamer solution using the HPLC system through the Schiff-base activation chemistry. In the Schiff-base activation, the monoliths were interacted with 15 mL of EDA at 60 °C for 12 h, rinsed with deionised water to remove any residual EDA, and exposed to 15 mL of 10% GA solution at 25 °C. The glutaraldehyde functionalised monoliths were equilibrated with buffer A followed by 20 µM aptamer covalent immobilisation at 0.2 mL/min. Aptamer-modified disk-monoliths (macroporous disk-aptasensors) were later washed with buffer A to remove non-specifically bound aptamer molecules. 5 mg/mL NaBH_3_CN solution was used in capping unreacted epoxy rings for 1 h followed by washing with the mobile phase buffer B, (10 mM Tris HCl + 5 mM MgCl_2_). Thrombin solution was prepared in buffer B and was used to determine the binding affinity of the monolith by chromatography.

#### FTIR and SEM characterisations

Analysis of surface morphology was carried by Scanning Electron Microscopy or SEM (Model S-3400N, Hitachi, Japan) after drying the polymethacrylate disk-monoliths at 60 °C for 24 h. The monolith surface was sputter-coated with gold to enable signal conduction. Fourier Transform Infra-Red spectroscopy or FTIR (Agilent Cary 630 FTIR, USA) was used to identify the newly introduced functional moieties into the polymer matrix. The FTIR analysis was conducted for both the blank and the aptamer-functionalised monoliths.

#### Thermogravimetric analysis and differential scanning calorimetry

Thermogravimetric analyses (TGA) of polymethacrylate monolith, Schiff base activated monolith, and aptamer immobilised monolith were carried out under an inert condition with a N_2_ gas flow rate of 25 cm^3^/min. The samples were exposed to a dynamic heating rate of 10 °C/min from 25 °C to 500 °C using DSC/TGA Mettler Toledo device. The same temperature range was used for DSC characterisation. Samples were analysed in triplicate for each experiment.

#### Zeta potential analysis of functionalised polymethacrylate monoliths

Different ionic concentrations of NaCl and MgCl_2_ ranging from 0–3.5 M were prepared to investigate the effect of ionic strength on the zeta potential of the aptamer-modified monoliths. The aptamer-modified monolith was first pulverised uniformly and conditioned with the salt concentrations for about 20 min, and the zeta potential measurements were carried out. The effects of pH on the charge distribution was also studied in a similar way. Zeta potential measurements were taken using Malvern Nano ZS, equipped with a folded capillary cell to hold sample solutions. Samples were analysed in triplicate for each experiment.

#### Liquid chromatographic analysis

An NGC Discover HPLC system (Next Generation Chromatography Discover 100 Chromatography system, BIORAD, Melbourne, Australia) was used for the liquid chromatographic studies. This system is equipped with two F10 system pumps, which are capable of achieving a flow rate of 10 mL/min at 25.2 MPa, multi-wavelength detector module, F100 sample pump for a maximum flow rate of 100 mL/min at 10 MPa, an inlet valve, mixer, column switching valve, sample inject valve, buffer blending valve, pH valve, communication adaptor, ChromLab software, and an integrated system touch screen. The chromatographic column adopted was a BIORAD polypropylene column (Econo-Pac Chromatography columns, 12 cm × 1.5 cm i.d) attached with an adjustable flow adaptor (Econo-Pac Flow Adaptor, catered for 1.5 cm column i.d). The BIORAD polypropylene columns contained 0.5 mL of the developed aptamer-modified disk-monolith connected to an adjustable flow adaptor and configured to the HPLC system. The feed mobile phase containing thrombin was prepared using buffer B (10 mM Tris HCl + 5 mM MgCl_2_), under different ionic and hydrogen potential conditions. The applied flow rate for the mobile phase was 0.3 mL/min under different ionic (0, 5, 10, 15, 20 mM MgCl_2_) and pH (4, 6, 7, 8, 10) conditions. The mobile phase was loaded for 18 mins using buffer B prior to elution. Elution of bound thrombin was done with 2 M NaClO_4_ with subsequent washing with buffer B.

## Results and Discussion

### Molecular dynamics simulation

**Figure 1:**
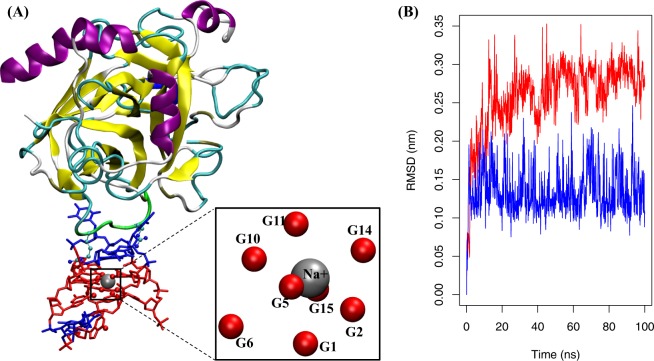
(**A**) A snapshot of the thrombin-aptamer complex after 100 ns MD simulation. The thrombin protein is shown in cartoons and the TBA in sticks. Guanines are plotted in red and Thymines in blue. Two positively charged Arginine (sticks and spheres) residues from a u-pin segment (residues R68 to N74, in green) directly interact with the TBA during the MD simulation. A sodium cation (grey sphere) is sandwiched by two G4’s during the MD simulation through interactions with the O6 atoms of the two G4’s (inset). (**B**) Comparison of the RMSD profiles of the thrombin-aptamer complex (blue) and an apo-form aptamer without stabilization by the Na^+^ cation and/or thrombin (red).

To gain a structural perspective of the TBA affinity binding, a model was built using the X-ray crystallographic structure of a complex between human α-thrombin and the 15-mer TBA (PDB entry 4DIH)^34^. During the 100 ns MD, two Guanine-quadruplexes (G4) were stabilized by both a sodium cation and the thrombin protein **(**Fig. 1A**)**. Under the same conditions, we further performed MD simulation on an apo-TBA system without the thrombin protein and sodium cation. For both systems, we monitored the root-mean square deviation (RMSD) of the O6 atoms of all bases in the TBA, which form electrostatic interactions with the sodium cation **(**Fig. 1A, insert) from the two G4s, and it was shown that significantly bigger RMSD were observed in the MD simulation of the apo-TBA, compared to the thrombin-TBA system **(**Fig. 1B**)**, suggesting a strong stabilization effect of the protein and/or Na^+^ cation to maintain the G4 structures in the TBA.

### SEM and FTIR analyses

**Figure 2:**
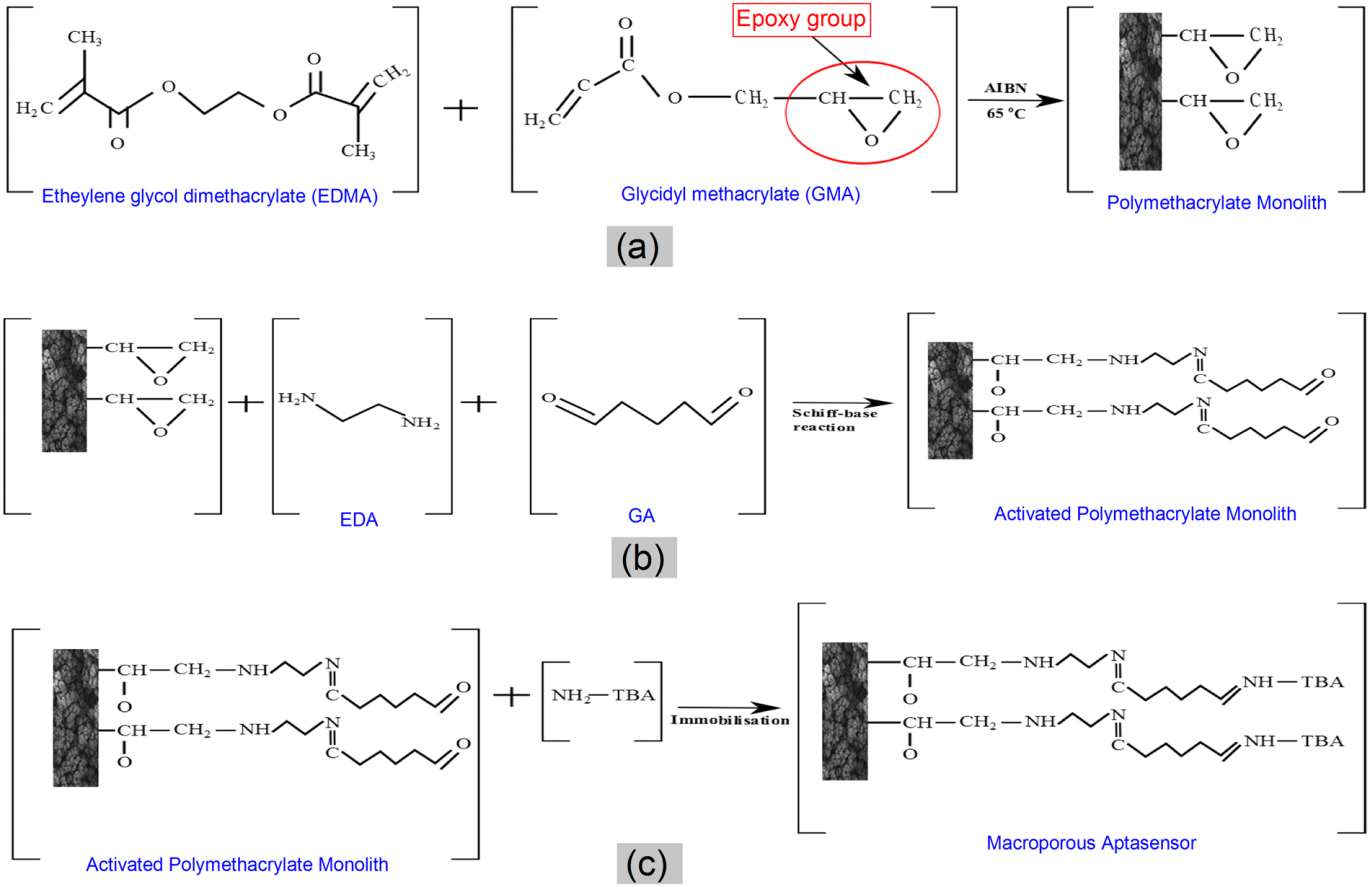
Schematic presentation of molecular interactions governing the development of the aptamer-modified disk-monolith. (**a**) Synthesis of polymethacrylate monoliths. (**b**) Activation of polymethacrylate monolith via Schiff-base chemistry. (**c**) Covalent immobilisation of amine-modified thrombin binding aptamers (TBA).

Polymethacrylate monoliths are continuous polymers, which can be synthesised into various shapes and forms depending on their mould. They enable smooth functionalisation with chemical and biomolecular ligands, and possess favorable, convective mass transport characteristics. Disk-monoliths have been previously demonstrated to attain remarkably enhanced hydrodynamic flow properties with minimal backpressure^35–37^. To achieve a covalently bonded interaction between the TBA and the monolith, a Schiff-base activation chemistry was employed, as shown in the reaction scheme in Fig. 2.

**Figure 3:**
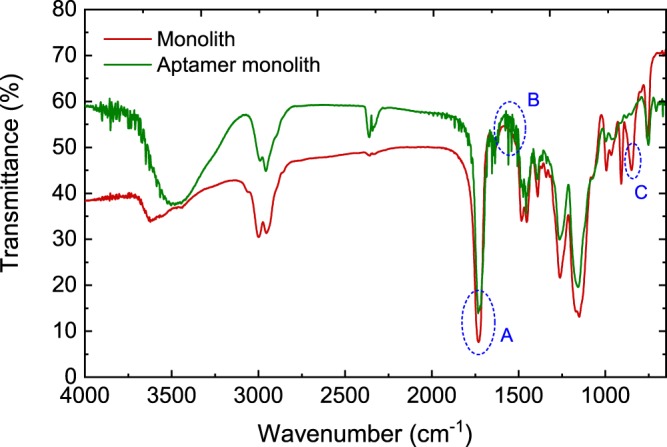
FTIR spectroscopic characterisation of original poly(GMA-co-EDMA) and aptamer-functionalised poly(GMA-co-EDMA) monoliths over a wavenumber range: 4000–600 cm^−1^. Three distinct events could be identified: (**A**) C=O stretch from the ester group of the polymethacrylate (~1718 cm^−1^), (**B**) heterocyclic compounds (~1500–1600 cm^−1^), and (**C**) Epoxy group (~847 cm^−1^).

The covalent bond established between the aptamer and monolith minimizes potential leaching of aptamers during multiple applications. In Fig. 3, the FTIR spectrographs of the original and aptamer-functionalised monoliths show the absence of epoxy moieties in the aptamer-functionalised monolith at ~847 cm^−1^. The original monolith showed the presence of epoxy functional groups.

**Figure 4:**
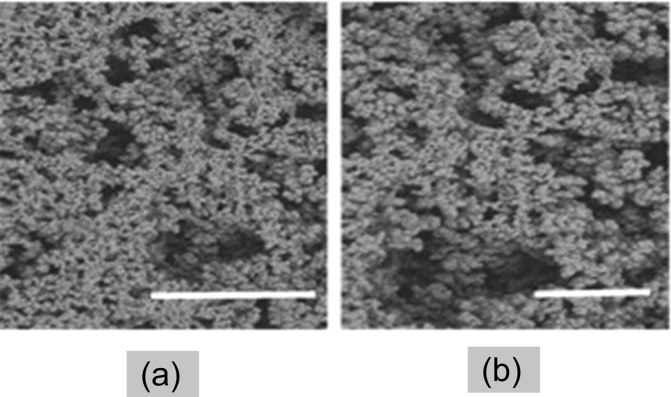
SEM images of polymethacrylate monoliths at magnifications of (**a**) x2000 with pore size of 20 µm and (**b**) x3000 with pore size of 10 µm.

Reaction of the epoxy moieties with ethylenediamine creates active sites for molecular interactions with glutaraldehyde. The reaction between the amine and epoxy moieties is based on a nucleophilic attack. The subsequent reaction with glutaraldehyde introduces a covalent linkage between the amine-activated monolith and the C6-amine-modified aptameric ligand. Since aptamers are synthetic nucleic acids, the occurrence of spectrograph peaks (between 1500–1600 cm^−1^) corresponding to the aptamer-functionalised monolith, being indicative of heterocycle compounds, further demonstrate successful coupling of aptameric ligands onto the monolith. It has been reported that the nature and type of porogenic solvent affect the physiochemical characteristics and surface morphology of polymethacrylate monoliths^32,38^, and this, in turn, influences the molecular arrangement of the pore surface, as well as the degree of ligand immobilisation. Consequently, in order to optimise the ligand density of the aptamer-modified disk-monolith, a microporogen (cyclohexanol) was used to enhance the monolith permeability. This, without compromising on the effective pore surface area for ligand immobilisation. The ligand density of the aptamer-modified disk-monolith was estimated using Eq. 1,

1 $$q=\frac{({C}_{o}-C){V}_{s}}{{V}_{m}},$$

where *q* is the ligand density of the aptamer (mol/L) covalently immobilized on the monolith; *C*_*o*_ and *C* are the initial and final concentration of aptamer solution (mol/L), respectively; and *Vs* and *V*_*m*_ are the volume (mL) of the aptamer solution and the monolith, respectively. The quantified ligand density, estimated to be 480 pmol/µL, bears the same order of magnitude when compared to the previously reported ligand densities in literature: 170 pmol/µL^24^, 204 pmol/µL^39^, 290 pmol/µL^23^, and 568 pmol/µL^40^. However, applications of different techniques and porous supports should not be overlooked in this regard. Figure 4 shows the SEM images, with heterogeneous, pore surface interconnections, and morphology of the disk-monolith, synthesised at 65 °C in a cyclohexanol microporogen.

### Thermogravimetric and differential scanning calorimetry analyses

**Figure 5:**
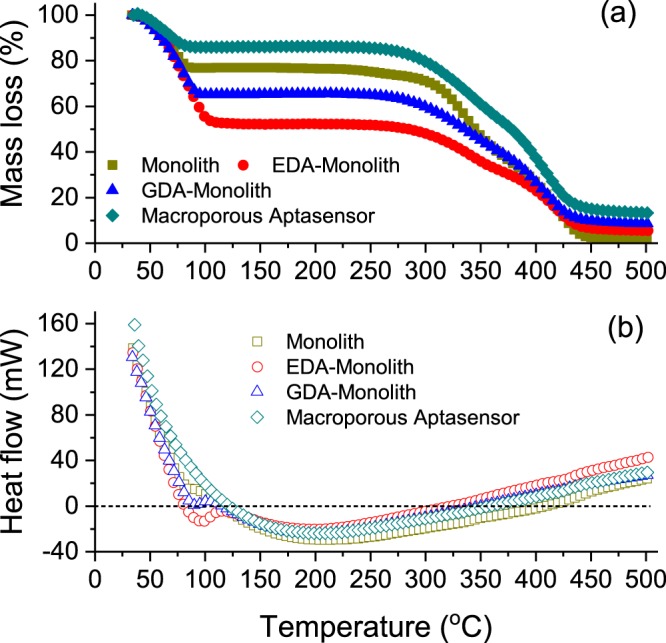
(**a**) Thermogravimetric analysis of polymethacrylate monolith, EDA-activated polymethacrylate monolith, GDA functionalised polymethacrylate monolith, and C6-aptamer-modified monolith. (**b**) Heat flow curves as obtained from DSC for the aptamer-modified monolith (macroporous aptasensor) development via Schiff-base covalent chemistry. Data points represent the average of 3 replicates (n = 3).

Thermogravimetric analysis of the aptamer-modified disk-monolith is important to probe the effects of temperature on the stability of the molecular interactions between the aptameric ligand and the polymer. Understanding the effects of synthesis and physicochemical conditions on the stability of polymethacrylate monoliths is critical to the development of robust aptamer-modified monoliths for mass and routine applications. The polymethacrylate monolith was fabricated through a thermally-initiated, free-radical polymerisation process, involving a functional monomer (GMA) and a cross-linker (EDMA) in a porogen. Using DSC analysis, Mihelič *et al*.^41^, demonstrated that the administration of initiators reduces the activation temperature necessary for the initiation of the polymerisation process. Figure 5 shows the TG and DSC patterns for each step, during the development of the aptamer-modified monolith. The average thermal degradation curve, for each process condition, was observed to possess a similar pattern to that of the original polymethacrylate monolithic adsorbent. The differences in mass loss, from 25–100 °C for the polymer samples, were due to differences in moisture contents after air drying. This is in agreement with previously reported TGA characterisations of polymers^33,42^. Past studies have shown that depending on the degree of cross-linking, polymethacrylate resin degradation commenced within the temperature range of 191–210 °C^33,43,44^. As shown in Table 1, noticeable increments were noted in the degradation temperature for each process step, which is in compliance with the findings reported elsewhere^45^.

**Table 1:** Characterisation of Schiff-base activated-monoliths via TGA, for aptamer immobilisation.

Sample	Onset of Degradation (°C)	Total Residual Mass%	DSC Endothermic Peak (mW)	DSC Peak Temp (°C)
*Monolith*	196.0 (±1.8)	0.250 (±1.82)	−41.8 (±11.1)	200.1 (±6.5)
*EDA-Monolith*	235.9 (±6.1)	0.538 (±0.036)	−25.9 (±8.4)	209.6 (±5.9)
*GA-Monolith*	255.4 (±2.7)	0.545 (±0.034)	−23.7 (±4.7)	215.3 (±6.3)
*Aptamer-modified monolith*	273.7 (±2.5)	0.653 (±0.076)	−23.05 (±1.4)	220.2 (±1.1)

Entries correspond to the mean and standard deviations of 3 replicates.

This phenomenon can be attributed to the formation of covalent bonds as a consequence of either the Schiff-base activation or aptamer immobilisation processes. Throughout this process, the amine functional groups of ethylenediamine and the amine-modified C6-aptamer, nucleophilically confront the epoxy functionalities of the polymethacrylate monolith. This leads to the substitution of C-O bonds of the epoxy moieties within the polymethacrylate matrix, with C=N bond during the amine reductive process. Functionalisation of the monolith with covalent chemistries, using ethylenediamine and glutaraldehyde activation, further increases the degree of cross-linking and mechanical properties, thereby enhancing the thermal stability^45,56^. Additionally, the incorporation of stable, short chain nucleotides from the aptamer onto the polymer matrix can enhance the thermal stability of the complex. The total residue for each functionalised and immobilised sample increased after complete degradation, due to an increase in the organic content per unit of the sample mass. This observation shows that effective activation and immobilisation of the polymethacrylate resins, is linked with an increase in thermal stability.

Heat flow curves, as obtained from DSC, showed significant variations in the peak heat energies of the samples, indicating the effectiveness of the grafting process. Endothermic peak heat energies increased after covalent reactions of ethylenediamine, glutaraldehyde and aptamer immobilisation, due to the enhancement in the degree of cross-linking. Furthermore, there was a shift in peak temperatures towards the right concurrently with the increase in endothermic peak heat energies.

### Chromatographic performance of aptamer-based disk monolith

The characteristic chromatographic parameters of the aptamer-based disk monolith were determined and shown in Table 2. Chromatographic sensors with a resolution value ≥1.5 are desirable for high-throughput affinity binding and separation. The column efficiency obtained by virtue of the number of theoretical plates was 109.2, although this can be improved by optimising the flow rate^26,46^. Binding performance of affinity-based separation devices are affected by factors such as buffer solution, ionic content, pH, biomolecular probe, polymeric adsorbent and flow rate^7,47^.

**Table 2:** Chromatographic parameters of aptamer-based disk monolith.

Parameter	Value
Column resolution (R_c_)	7.55
Retention factor (R_f_)	4.19
Number of theoretical Plates (N)	109.2
Capacity factor (k)	3.79
Peak asymmetry (A_s_)	1.86
Selectivity factor (α)	1.44

#### Effect of monovalent and divalent cations on zeta potential and chromatographic performance

The secondary structure of aptamers has been shown to be affected by the presence of monovalent and divalent ions resulting in conformational changes. As the binding mechanisms of aptamers may vary with systems^48^, investigating the ionic concentration in a binding medium, is necessary to optimise the binding kinetics and performance of the aptamers to their cognate targets in a chromatographic sensor.

**Figure 6:**
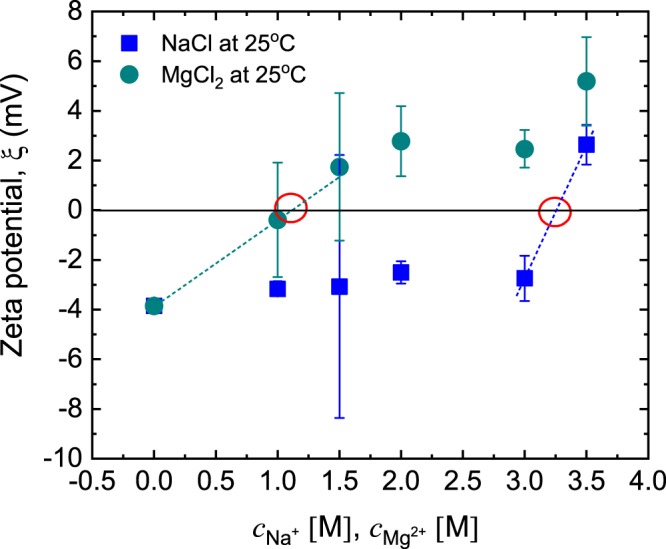
Effect of [Na^+^] and [Mg^2+^] on the zeta potential of pulverised aptamer-modified monoliths conducted at 25 °C using a folded capillary cell. Three replicates were recorded for each data point. The dashed lines represent least-squares linear fits to the data, for estimating the pI using linear interpolation between the nearest data points, in either case.

The zeta potentials of the aptamer-functionalised adsorbents were analysed based on their electrophoretic mobility after pulverization and incubation, under varying process conditions of pH and ionic strength. The thrombin binding DNA aptamers possess G-quadruplex structures, with conformations and stability that can be altered by ionic compositions and concentrations of their microenvironment^49,50^. In previous reports, the effects of different cations on aptamer structural integrity were studied, via circular dichroism spectroscopy (CDS), nuclear magnetic resonance (NMR), quartz crystal microbalance (QCM) and X-ray photoelectron spectroscopy (XPS)^18,50–52^. Hianik *et al*.^18^ studied the effect of Na^+^ ions on immobilised aptasensors via QCM. They observed that although Na^+^ had minimal effects on the stability of G-quadruplex conformation, a gradual increase in concentration resulted in a corresponding decrease in the aptamer sensitivity due to shielding effects. To further understand the influence of cationic species on the charge stability and binding sensitivity, different concentrations of Na^+^ and Mg^2+^ ions were interacted with the pulverised aptamer-modified monolith and the resulting zeta potentials were analysed. As shown in Fig. 6, increasing the ionic concentration of Mg^2+^ resulted in a more rapid decrease in the electronegativity of the aptamer. Mg^2+^ has a smaller ionic radius (1.5 Å) and higher electropositive charge compared to Na^+^ (1.8 Å). This results in a higher charge density for Mg^2+^ that creates improved shedding effect at the aptamer anionic interface. The aptamer isoelectric focussing points, i.e., pI, in the presence of Na^+^ and Mg^2+^ ions, were established at concentrations of 1.12 M for Mg^2+^ and 3.25 M for Na^+^.

**Figure 7:**
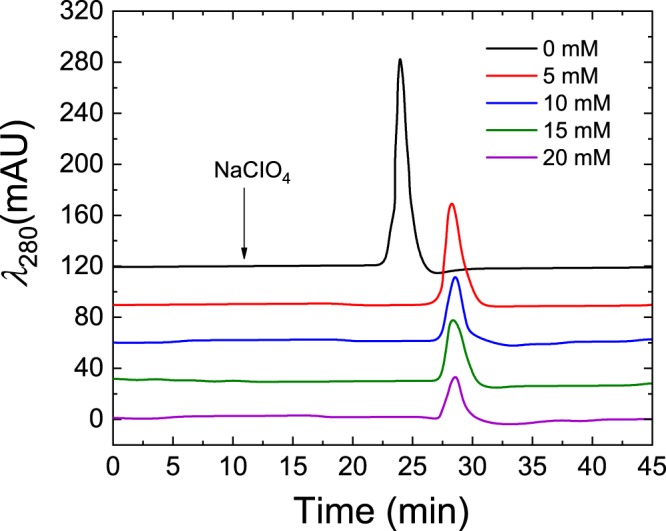
Effect of [Mg^2+^] on chromatographic binding characteristics of thrombin. The chromatograph shows decreasing thrombin concentration with increasing [Mg^2+^]. The chromatograph was generated by HPLC at a mobile phase flow rate of 0.3 mL/min and eluted with 2 M NaClO_4_ after 18 min of sample loading.

The effects of Mg^2+^ on the chromatographic characteristics of the aptamer-modified disk-monolith were studied over an ionic concentration range of 0–20 mM in the mobile phase buffer at pH = 8.0. Generally, an increase in the ionic strength of the mobile phase resulted in increasing retention time of the thrombin molecule, as follows: 0 M (23.96 min); 5 mM (28.23 min); 10 mM (28.42 min); 15 mM (28.36 min) and 20 mM (28.32 min). This is shown in Fig. 7 and is attributed to the induced synthesis of G-quadruplex secondary structure of the thrombin binding aptamer (TBA), and its subsequent stabilisation by Mg^2+^ ^48,53,54^. Increasing concentrations of monovalent and divalent ions affect the helix-helix electrostatic interactions in the polynucleotide aptamer^55^. The inherent polyanionic nature of aptamers, due to their phosphodiester backbone, renders them as negatively charged molecules with an anisotropic distribution^53^. The anisotropic distribution and high electronegativity are therefore minimised by the introduction of either Mg^2+^ or any other suitable divalent cations, or high concentrations of monovalent cations, for stabilising the aptamer^53,54^. Also, increasing [Mg^2+^] introduces positive electrostatic binding sites on the aptamer-modified disk-monolith to increase thrombin molecular retention. This leads to a reduction in the concentration of thrombin eluted under the constant elution program. Notably, the zero [Mg^2+^] mobile phase buffer showed the highest thrombin concentration in the elution, with the shortest retention time, demonstrating the absence of extra positive electrostatic binding sites that increase retention. It should be noted that increasing [Mg^2+^] may compromise the binding specificity of the aptamer-modified disk-monolith, resulting in increasing electrostatic binding of the electronegative, non-targeted species. Also, Mg^2+^ ions shield active electronegative sites of the aptamer, making them unavailable or less sensitive towards thrombin binding.

#### Effect of pH on charge distribution and chromatographic performance

**Figure 8:**
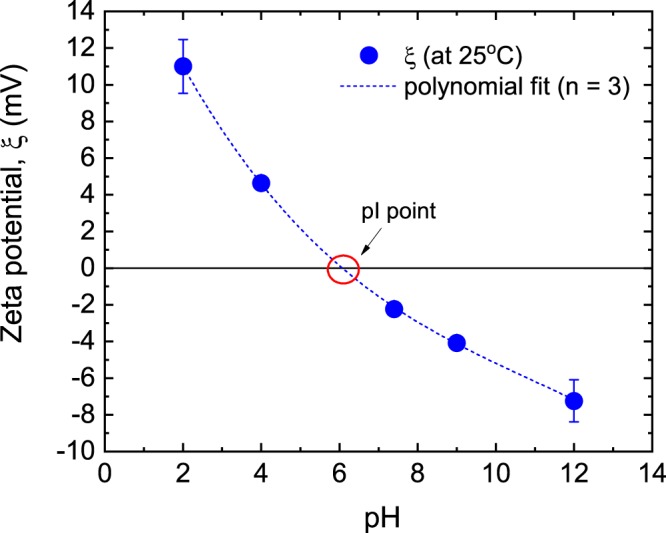
Effect of pH on the zeta potential of the pulverised aptamer-modified monolith conducted at 25 °C using a folded capillary cell. The dashed line represents a least-squares cubic polynomial curve fit to the data. pI was estimated through interpolation.

The effect of pH on the charge distribution of the aptamer-modified disk-monolith was investigated after pulverising the aforementioned device, and conditioning them with different volumes of either 0.01 M HCl or 0.01 M NaOH in PBS buffer, to establish a pH range. Since the monolith was immobilised with TBA in PBS buffer at pH 7.4, with an original zeta potential of −2.37 mV (±0.38), this condition was used as the baseline. Figure 8 shows the effect of pH variation on the net charge of the aptamer-modified disk-monolith.

The zeta potential of the aptamer-modified monolith decreased from 11 mV to −7.2 mV with an increase in pH from 2.0 to 12. This trend could be attributed to a two-step phenomenon: (i) adsorption of H^+^ and OH^−^ on the surface of the aptamer-modified monolithic particulates, and (ii) protonation and deprotonation of aptamer-modified monolith moieties under decreasing or increasing pH, respectively. Since the aptamers are single stranded nucleic acids, they possess moieties that can undergo ionisation. Notably, carbonyl and phosphate moieties of the aptamer are known to undergo deprotonation in the presence of acids, whereas pyrimidines (cytosine, thymine and uracil) are susceptible to protonation, resulting in variations in zeta potential magnitudes.

**Figure 9:**
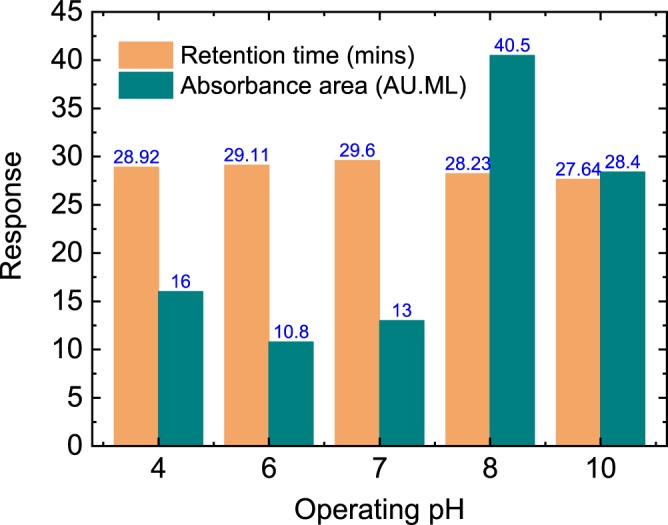
Effect of operating pH on the retention time and the peak absorbance area of thrombin detection. The chromatograph was generated by HPLC at a mobile phase flow rate of 0.3 mL/min, and eluted with 2 M NaClO_4_ after 18 mins of sample loading.

Figure 9 shows the effect of pH on retention time and absorbance areas of thrombin peaks. The neutral operating pH showed the longest retention time with a relatively low thrombin concentration eluted. However, the alkaline regions were observed to have higher concentrations of thrombin in the eluted fractions. Consequently, the optimum operating pH of the mobile phase was determined as 8.0. The pI of the target thrombin molecule is in the region of 6.35–7.6. This indicates the importance of the mobile phase pH in optimising the binding characteristics of the aptamer-modified disk-monolith, based on the pI of the target molecule.

## Conclusions

The current work demonstrates that the binding performance of free and immobilised aptamers on polymethacrylate monoliths, is affected by the physicochemical conditions in their micro-environment, including ionic concentration, pH and the chemistry of the supporting system. Thermogravitmetric analysis of the polymer activation and immoblisation showed increasing initial temperatures of degradation, corresponding to an increase in the amount of endothermic energy needed to degrade samples from each modification step. The use of microporogen in the synthesis of polymethacrylate monolith enhanced the surface area, resulting in a high aptameric ligand density of 480 pmol/uL. Data for thrombin retention and elution analysis showed an optimal operating condition of pH = 8.0 and [Mg^2+^] = 5 mM for TBA-thrombin interaction and polymeric adsorption. This body of work is critical in understanding biophysical activities, governing aptamer-thrombin interactions at the polymer pore surface, and their significant impacts on chromatographic performance.

## Data Availability

All data generated or analyzed during this study are included in this manuscript.

## References

[1] Velusamy V, Arshak K, Korostynska O, Oliwa K, Adley C (2010). An overview of foodborne pathogen detection: In the perspective of biosensors. Biotechnol. Adv..

[2] Kirsch J, Siltanen C, Zhou Q, Revzin A, Simonian A (2013). Biosensor technology: recent advances in threat agent detection and medicine. Chem. Soc. Rev..

[3] Pakchin PS, Nakhjavani SA, Saber R, Ghanbari H, Omidi Y (2017). Recent advances in simultaneous electrochemical multi-analyte sensing platforms. TrAC Trends Anal. Chem..

[4] Lopez-Barbosa N, Gamarra JD, Osma JF (2016). The future point-of-care detection of disease and its data capture and handling. Anal. Bioanal. Chem..

[5] Mascini M, Tombelli S (2008). Biosensors for biomarkers in medical diagnostics. Biomarkers.

[6] Acquah C (2015). Deploying aptameric sensing technology for rapid pandemic monitoring. Crit. Rev. Biotechnol..

[7] Acquah, C., Danquah, M. K., Yon, J. L. S., Sidhu, A. & Ongkudon, C. M. A review on immobilised aptamers for high throughput biomolecular detection and screening. *Anal*. *Chim*. *Acta* **888** (2015). 10.1016/j.aca.2015.05.050 26320953

[8] Chen A, Yang S (2015). Replacing antibodies with aptamers in lateral flow immunoassay. Biosens. Bioelectron..

[9] Tan, S. Y. *et al*. SELEX Modifications and Bioanalytical Techniques for Aptamer–Target Binding Characterization. *Crit*. *Rev*. *Anal*. *Chem*. **46** (2016). 10.1080/10408347.2016.1157014 26980177

[10] Schulz C (2016). Generating Aptamers Interacting with Polymeric Surfaces for Biofunctionalization. Macromol. Biosci..

[11] Robertson DL, Joyce GF (1990). Selection *in vitro* of an RNA enzyme that specifically cleaves single-stranded DNA. Nature.

[12] Tuerk C, Gold L (1990). Systematic evolution of ligands by exponential enrichment: RNA ligands to bacteriophage T4 DNA polymerase. Science (80−.)..

[13] Ellington AD, Szostak JW (1990). *In vitro* selection of RNA molecules that bind specific ligands. Nature.

[14] Javaherian S, Musheev MU, Kanoatov M, Berezovski MV, Krylov SN (2009). Selection of aptamers for a protein target in cell lysate and their application to protein purification. Nucleic Acids Res..

[15] Nezlin R (2016). Use of aptamers in immunoassays. Mol. Immunol..

[16] Ilgu M, Nilsen-Hamilton M (2016). Aptamers in analytics. Analyst.

[17] Monaco I (2017). Aptamer Functionalization of Nanosystems for Glioblastoma Targeting through the Blood–Brain Barrier. J. Med. Chem..

[18] Hianik T, Ostatná V, Sonlajtnerova M, Grman I (2007). Influence of ionic strength, pH and aptamer configuration for binding affinity to thrombin. Bioelectrochemistry.

[19] Oktem HA, Bayramoglu G, Ozalp VC, Arica MY (2007). Single-Step Purification of Recombinant Thermus aquaticus DNA Polymerase Using DNA-Aptamer Immobilized Novel Affinity Magnetic Beads. Biotechnol. Prog..

[20] Jiang X (2017). Electrochemiluminescence Biosensor Based on 3-D DNA Nanomachine Signal Probe Powered by Protein-Aptamer Binding Complex for Ultrasensitive Mucin 1 Detection. Anal. Chem..

[21] Su Z (2017). Effective covalent immobilization of quinone and aptamer onto a gold electrode via thiol addition for sensitive and selective protein biosensing. Talanta.

[22] Brothier F, Pichon V (2014). Miniaturized DNA aptamer-based monolithic sorbent for selective extraction of a target analyte coupled on-line to nanoLC. Anal. Bioanal. Chem..

[23] Han B, Zhao C, Yin J, Wang H (2012). High performance aptamer affinity chromatography for single-step selective extraction and screening of basic protein lysozyme. J. Chromatogr. B.

[24] Zhao Q, Li X-F, Shao Y, Le XC (2008). Aptamer-Based Affinity Chromatographic Assays for Thrombin. Anal. Chem..

[25] Du K, Yang M, Zhang Q, Dan S (2016). Highly Porous Polymer Monolith Immobilized with Aptamer (RNA) Anchored Grafted Tentacles and Its Potential for the Purification of Lysozyme. Ind. Eng. Chem. Res..

[26] Acquah Caleb, Danquah Michael K., Chan Yi Wei, Moy Charles K. S., Ongkudon Clarence M., Lau Sie Yon (2018). Chromatographic characterisation of aptamer-modified poly(EDMA-co-GMA) monolithic disk format for protein binding and separation. Separation Science and Technology.

[27] Acquah, C., Moy, C. K. S. C. K. S., Danquah, M. K. M. K. & Ongkudon, C. M. C. M. Development and characteristics of polymer monoliths for advanced LC bioscreening applications: A review. **1015**–**1016**, 121–134 (2016). 10.1016/j.jchromb.2016.02.016 26919447

[28] Roberts MWH, Ongkudon CM, Forde GM, Danquah MK (2009). Versatility of polymethacrylate monoliths for chromatographic purification of biomolecules. J. Sep. Sci..

[29] Phillips JC (2005). Scalable molecular dynamics with NAMD. J. Comput. Chem..

[30] Vanommeslaeghe K (2009). CHARMM general force field: A force field for drug-like molecules compatible with the CHARMM all-atom additive biological force fields. J. Comput. Chem..

[31] Price DJ, Brooks CL (2004). A modified TIP3P water potential for simulation with Ewald summation. J. Chem. Phys..

[32] Ongkudon CM, Danquah MK (2010). Process optimisation for anion exchange monolithic chromatography of 4.2kbp plasmid vaccine (pcDNA3F). J. Chromatogr. B.

[33] Acquah, C., Danquah, M. K., Moy, C. K. S., Anwar, M. & Ongkudon, C. M. Thermogravimetric characterization of *ex situ* polymethacrylate (EDMA-co-GMA) monoliths. *Can*. *J*. *Chem*. *Eng*. **95** (2017).

[34] Russo Krauss I (2012). High-resolution structures of two complexes between thrombin and thrombin-binding aptamer shed light on the role of cations in the aptamer inhibitory activity. Nucleic Acids Res..

[35] Barut M, Podgornik A, Brne P, Štrancar A (2005). Convective Interaction Media short monolithic columns: Enabling chromatographic supports for the separation and purification of large biomolecules. J. Sep. Sci..

[36] Trauner A, Bennett MH, Williams HD (2011). Isolation of Bacterial Ribosomes with Monolith Chromatography. PLoS One.

[37] Kramberger P, Honour RC, Herman RE, Smrekar F, Peterka M (2010). Purification of the Staphylococcus aureus bacteriophages VDX-10 on methacrylate monoliths. J. Virol. Methods.

[38] Danquah MK, Forde GM (2008). Preparation of macroporous methacrylate monolithic material with convective flow properties for bioseparation: Investigating the kinetics of pore formation and hydrodynamic performance. Chem. Eng. J..

[39] Deng Q, German I, Buchanan D, Kennedy RT (2001). Retention and Separation of Adenosine and Analogues by Affinity Chromatography with an Aptamer Stationary Phase. Anal. Chem..

[40] Kökpinar Ö, Walter J-G, Shoham Y, Stahl F, Scheper T (2011). Aptamer-based downstream processing of his-tagged proteins utilizing magnetic beads. Biotechnol. Bioeng..

[41] Mihelič I, Krajnc M, Koloini T, Podgornik A (2001). Kinetic Model of a Methacrylate-Based Monolith Polymerization. Ind. Eng. Chem. Res..

[42] Vlad CD, Dinu MV, Dragan S (2003). Thermogravimetric analysis of some crosslinked acrylamide copolymers and ion exchangers. Polym. Degrad. Stab..

[43] Acquah, C., Danquah, M. K., Moy, C. K. S., Anwar, M. & Ongkudon, C. M. Parametric investigation of polymethacrylate monolith synthesis and stability via thermogravimetric characterisation. *Asia-Pacific J*. *Chem*. *Eng*. **12** (2017).

[44] Yusuf K, Badjah-Hadj-Ahmed AY, Aqel A, ALOthman ZA (2016). Monolithic metal-organic framework MIL-53(Al)-polymethacrylate composite column for the reversed-phase capillary liquid chromatography separation of small aromatics. J. Sep. Sci..

[45] Awad, G. E. A., Abd El Aty, A. A., Shehata, A. N., Hassan, M. E. & Elnashar, M. M. Covalent immobilization of microbial naringinase using novel thermally stable biopolymer for hydrolysis of naringin. *3 Biotech* **6** (2016). 10.1007/s13205-015-0338-x PMC4703588 28330084

[46] Wang S, Li X, Sun Y (2019). Poly(N,N-dimethylaminopropyl acrylamide)-grafted Sepharose FF: A new anion exchanger of very high capacity and uptake rate for protein chromatography. J. Chromatogr. A.

[47] Razdan, S., Wang, J. C. & Barua, S. PolyBall: A new adsorbent for the efficient removal of endotoxin from biopharmaceuticals. *Sci*. *Rep*. **9** (2019). 10.1038/s41598-019-45402-w PMC6586805 31222053

[48] Lin P-H (2008). Microcalorimetrics Studies of the Thermodynamics and Binding Mechanism betweenl-Tyrosinamide and Aptamer. J. Phys. Chem. B.

[49] Cho M-S (2008). Detection for folding of the thrombin binding aptamer using label-free electrochemical methods. BMB Rep..

[50] Nagatoishi S, Tanaka Y, Tsumoto K (2007). Corrigendum to “Circular dichroism spectra demonstrate formation of the thrombin-binding DNA aptamer G-quadruplex under stabilizing-cation-deficient conditions”. Biochem. Biophys. Res. Commun..

[51] Lin P-H (2011). Studies of the binding mechanism between aptamers and thrombin by circular dichroism, surface plasmon resonance and isothermal titration calorimetry. Colloids Surfaces B Biointerfaces.

[52] Ostatná V, Vaisocherová H, Homola J, Hianik T (2008). Effect of the immobilisation of DNA aptamers on the detection of thrombin by means of surface plasmon resonance. Anal. Bioanal. Chem..

[53] Noeske J, Schwalbe H, Wohnert J (2007). Metal-ion binding and metal-ion induced folding of the adenine-sensing riboswitch aptamer domain. Nucleic Acids Res..

[54] Tan, S. Y., Acquah, C., Tan, S. Y., Ongkudon, C. M. & Danquah, M. K. Characterisation of charge distribution and stability of aptamer-thrombin binding interaction. *Process Biochem*. **60** (2017).

[55] Tan Z-J, Chen S-J (2006). Ion-Mediated Nucleic Acid Helix-Helix Interactions. Biophys. J..

[56] Verma ML, Naebe M, Barrow CJ, Puri M (2013). Enzyme Immobilisation on Amino-Functionalised Multi-Walled Carbon Nanotubes: Structural and Biocatalytic Characterisation. PLoS One.

